# The First Offence

**Published:** 1867-10

**Authors:** 


### THE FIRST OFFENCE.

IN the cheerful dining-room of my friend Stephenson a select party was assembled to celebrate his birthday. A very animated discussion had been carried on for sometime as to whether the first deviation from integrity should be treated with severity or leniency. Various were the opinions, and various were the arguments brought forward to support them.

The majority appeared to lean to the side of "crush all offences in the bud," when a warm-hearted old gentleman exclaimed —

"Depend upon it, more young people are lost to society from the first offence being treated with injudicious severity, than from the contrary extreme. Not that I would pass over the slightest deviation from integrity, either in word or deed; that would certainly be mistaken kindness; but, on the other hand, neither would I punish with severity an offence committed, perhaps, under the influence of temptation—temptation, too, that we ourselves may have thoughtlessly placed in the way, in such a manner as to be irresistible."

"There is truth in what you say," remarked our benevolent host, who had hitherto taken no part in the conversation; "and it reminds me of a circumstance that occurred in the earlier part of my life, which may serve to illustrate the subject you have been discussing."

"In the outset of my business career," said he, "I took into my employment a young man as under clerk; and, according to a rule I had laid down, whenever a stranger entered my service his duties were of a nature to involve as little responsibility as possible, until sufficient time had been given to form a correct estimate of his character. This young man, whom I shall call Smith, was of a respectable family. He had lost his father, and had a mother and sisters in some measure dependent upon him.

"After he had been a short time in my employment, it happened that my confidential clerk, whose duty it was to receive the money from the bank for the payment of wages, being prevented by an unforeseen circumstance from attending at the proper time, sent the sum required by Smith.

"My confidence was so great in my head clerk that I was not in the habit of regularly counting the money when brought to me; but as on this occasion it had passed through other hands, I thought it right to do so. Therefore calling Smith back as he was leaving my counting-house, I desired him to wait a few minutes, and proceeded to ascertain if it was quite correct. Great was my surprise and concern on finding that there was a considerable deficiency.

"'From whom,' said I, 'did you receive this money?'

"He replied, 'From Mr.———,' naming my confidential clerk.

"'It is strange,' said I, 'but this money is incorrect.' He changed countenance, and his eye fell as I looked at him; but he answered, with considerable composure, 'that it was as he had received it.'

"After some further questioning I became convinced that the young man had taken the money.

"It is in vain,' I said at length, 'to impose upon me. I am convinced that you have taken this money, and that it is at this moment in your possession. The evidence against you is sufficient to justify me in immediately discharging you from my service. But you are a very young man; your conduct has, I believe, been hitherto correct, and I am willing to afford you an opportunity of redeeming the past. All knowledge of this matter rests between ourselves. Confess candidly, therefore, the error of which you have been guilty; restore what you have taken; endeavor, by your future good conduct, to deserve my confidence and respect, and this circumstance shall never transpire to injure you.'

"The poor fellow was deeply affected. In a voice almost inarticulate with emotion, he acknowledged his guilt, and said that, having frequently seen me receive the money without counting it, on being intrusted with it himself, the idea flashed across his mind that he might easily abstract some without incurring suspicion, or at all events, without there being sufficient evidence to justify it; that, being in distress, the temptation had proved stronger than his power of resistance, and he had yielded.

"'I cannot now,' he continued, 'prove how deeply your forbearance has touched me; time alone can show that it has not been misplaced.' He left me to resume his duties.

"'Days, weeks and months passed away, during which I scrutinized his conduct with the greatest anxiety, whilst at the same time I carefully guarded against any appearance of suspicious watchfulness; and with delight I observed that so far my experiment had succeeded. The greatest regularity and attention, the utmost devotion to my interests, marked his business habits; and this without any display; for his quiet and humble deportment was from that time remarkable.

"At length, finding his conduct invariably marked by openness and plain dealing, my confidence in him was so far restored that, on a vacancy occurring in a situation of greater trust and increased emolument than the one he had hitherto filled, I placed him in it; and never had I the slightest reason to repent of the part I acted towards him.

"For years he served me with fidelity and devotion. His character for rigid honesty was so well known, that 'as honest as Smith,' became a proverb among his acquaintances.

"One morning I missed him from his accustomed place, and learnt that he was detained at home by indisposition. Several days elapsed, and still he was absent; and upon calling at his house to inquire after him, I found the family in great distress on his account. His complaint had proved typus fever of a malignant kind. From almost the commencement of his attack he had, as his wife (for he had been for some time married,) informed me, lain in a state of unconsciousness, from which he had roused only to the ravings of delirium, and that the physician gave but little hopes of recovery.

"For some days he continued in the same state; at length a message was brought, saying that Mr. Smith wished to see me, the messenger adding, that Mrs. Smith hoped I would come as soon as possible, for she feared her husband was dying. I immediately obeyed the summons.

"On entering the chamber I found the whole of his family assembled to take farewell of him they so tenderly loved. As soon as he perceived me he motioned for me to approach near to him, and taking my hand in both of his, he turned towards me, full of gratitude and affection, and said,—

"'My dear master, my best earthly friend, I have sent for you that I may give you the thanks and blessings of a dying man for all your goodness to me. To your generosity and mercy I owe it that I have lived useful and respected, that I die lamented and happy. To you I owe it that I leave my children a name unsullied by crime, that in after years the blush of shame shall never tinge their cheeks at the memory of their father. O God,' he continued, 'Thou who hast said, "Blessed are the merciful," bless him. According to the measure he has meted out to others do thou mete out to him.'

"Then turning to his family, he said, 'My beloved wife and family, I intrust you without fear to the care of the Heavenly Parent who has said, "Leave thy fatherless children to Me, and I will preserve them alive, and let thy widows trust in me." And you, my dear master, will, I know, be to them as you have been to me—guide, protector and friend.'

"That," continued the old man, looking around upon us with glistening eyes, "though mixed with sorrow, was one of the happiest moments of my life. As I stood by the bed-side of the dying man, and looked upon his children growing up virtuous, intelligent, and respecting and honoring as much as they loved their father; when I saw his wife, though overcome with grief for the loss of a tender and beloved husband, yet sorrowing not as one without hope; when I saw him calmly waiting the inevitable stroke, trusting in the mercy of God, and at peace with his fellow men; and when I thought of what the reverse of all this might have been—crime, misery, a disgraceful and dishonored life, perhaps a shameful and violent death—had I yielded to the first impulse of indignation, I felt a happiness which no words can express.

"My friends, I am an old man. During a long and eventful career in business I have had intercourse with almost every variety of temper and disposition, and with many degrees of talent, but I have never found reason to swerve from the principles with which I set out in life 'to temper justice with mercy.'"

Such was the story of our friend. And I believe not one in that company but returned home more disposed to judge leniently of the failings of his fellow creatures, and, as far as lay in his power, to extend to all who might fall into temptation, that mercy which, under similar circumstances, he would wish shown to himself, feeling "that it is more blessed to save than to destroy."

